# Optimal tension of fascia lata graft with SuturePatch augmentation in superior capsule reconstruction for irreparable tears of the supraspinatus and infraspinatus tendons

**DOI:** 10.1016/j.jseint.2025.101611

**Published:** 2025-12-26

**Authors:** Teruhisa Mihata, Christen E. Chalmers, Joseph Carbone, Mauro Maniglio, Michael Künzler, Nilay A. Patel, Yukitaka Fujisawa, Michelle H. McGarry, Thay Q. Lee

**Affiliations:** a Department of Orthopaedic Surgery, Osaka-Jobe Sports Medicine & Orthopaedic Clinic, Osaka, Japan; b Orthopaedic Biomechanics Laboratory, Department of Orthopaedic Surgery, Creighton University School of Medicine, Omaha, NE, USA; c Department of Orthopedic Surgery, Katsuragi Hospital, Kishiwada, Osaka, Japan; d Department of Orthopaedics and Traumatology, Inselspital Bern, University Hospital, Bern, Switzerland; e Hand Surgical Department, The Balgrist, University Clinic Zurich, Zurich, Switzerland

**Keywords:** Augmentation, Biomechanics, Cadaver study, Superior capsule reconstruction, SuturePatch, Tension

## Abstract

**Background:**

Postoperative graft tear is correlated with poor clinical outcome after superior capsule reconstruction (SCR) for irreparable rotator cuff tears. The objective of this study was to investigate the optimal tension of the SuturePatch, which has been developed to reinforce SCR.

**Methods:**

Eight fresh-frozen cadaveric shoulders were tested by using a custom shoulder-testing system. Superior glenohumeral translation, subacromial peak contact pressure, and glenohumeral range of motion (ROM) were compared among 4 conditions: (1) intact; (2) irreparable supraspinatus and infraspinatus tears; (3) SCR using a thin fascia lata graft and SuturePatch augmentation performed at 20° glenohumeral abduction; and (4) SCR using the same graft with SuturePatch augmentation performed at 30° glenohumeral abduction.

**Results:**

Increased superior glenohumeral translation and subacromial peak contact pressure after creation of an irreparable supraspinatus and infraspinatus tendon tear significantly decreased after SCR with SuturePatch augmentation performed at both 20° and 30° glenohumeral abduction. Superior glenohumeral translation and subacromial peak contact pressure did not differ between 20° and 30° glenohumeral abductions. Internal rotation (*P* = .39-.99), external rotation (*P* = .61-.99), or total rotational ROM (*P* = .51-.99) were comparable between the irreparable supraspinatus and infraspinatus tendon tear condition and SCR with SuturePatch augmentation at both 20° and 30° glenohumeral abduction.

**Conclusion:**

SCR with SuturePatch augmentation restored superior glenohumeral stability and maintained glenohumeral ROM. When the SuturePatch is used to augment the graft of SCR, 30° or 45° of shoulder abduction (equal to 20° or 30° of glenohumeral abduction) may provide the optimal tension for graft attachment to the glenoid medially and greater tuberosity laterally.

For the treatment of irreparable rotator cuff tears, superior capsule reconstruction (SCR) was developed to restore superior glenohumeral stability and shoulder joint function.^12^, ^13^, ^14^, ^15^, ^16^, ^17^, ^18^, ^19^ Postoperative graft tear, which is correlated with poor clinical outcome,^16^, ^17^, ^18^ occurred in 5%-55% of SCR in previous studies.^5^^,^^10^^,^^11^^,^^16^, ^17^, ^18^ To improve the clinical outcome after SCR, we need to consider how to increase the graft healing rate.

A previous biomechanical study showed that graft thickness influences superior glenohumeral stability after SCR, and a graft thickness of 8 mm has been recommended.^13^ When a human dermal allograft is used for SCR, the thickness of graft is limited to 1-3 mm,^1^^,^^4^^,^^5^^,^^7^^,^^8^^,^^22^ which is insufficient to completely restore superior stability in the glenohumeral joint.^19^ Even when a fascia lata autograft is chosen for SCR, a graft thickness of 8 mm can be difficult to achieve in cases with thin fascia lata.

To address this limitation, recently, we developed SuturePatch (Arthrex, Naples, FL, USA) to reinforce thin grafts in SCR (Fig. 1). This mesh material can be shaped to cover the humeral head like a ‘hammock,’ similar to the description of the shoulder capsular ligaments in previous studies.^20^^,^^23^ Adding SuturePatch to a thin graft may improve superior stability after SCR. In addition, although SuturePatch is made of polyester, its mesh structure may allow physiological glenohumeral rotation without restricting shoulder motion. Because the tension applied to SuturePatch may influence both superior stability and range of motion (ROM) after SCR, determining the optimal tension is essential. The objective of this study was to identify the optimal tension of SuturePatch when reinforcing thin fascia lata grafts in SCR, and our hypothesis was that this optimal tension would not substantially deviate from the previously reported range of 10°-30° of glenohumeral abduction required to achieve appropriate graft tension in SCR without SuturePatch.^13^

**Figure 1:**
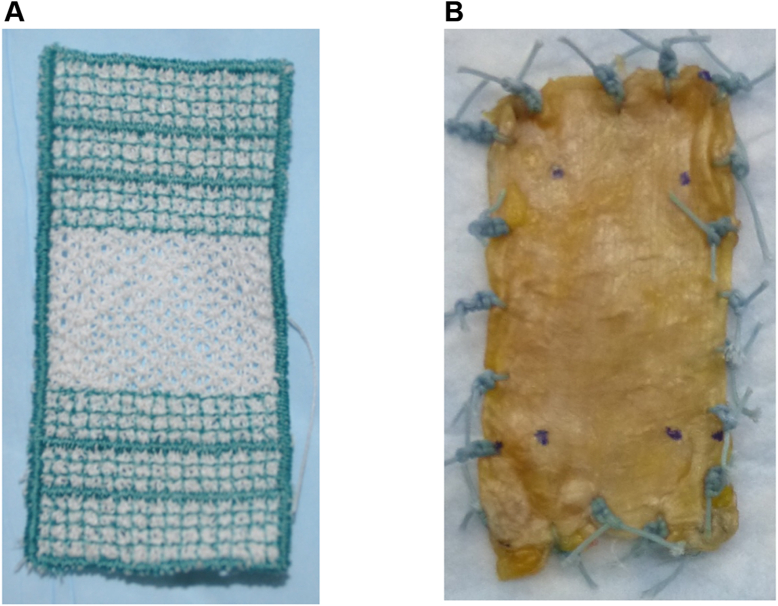
(**A**) SuturePatch. (**B**) Thin fascia lata graft with SuturePatch augmentation. The SuturePatch (thickness, 2 mm) was sandwiched between 2 layers of thin fascia lata (thickness [each layer], 1-2 mm).

## Materials and methods

### Specimen preparation and testing setup

Eight cadaveric shoulders from 6 male and 2 female donors (mean age, 62.6 years; range, 49-69 years) were used for this study. The fresh-frozen specimens were thawed overnight prior to dissection. All soft tissues were removed, except for the glenohumeral joint capsule, coracoacromial ligament, and tendinous insertions of the supraspinatus, infraspinatus, teres minor, subscapularis, deltoid (anterior, middle, and posterior), pectoralis major, and latissimus dorsi. All specimens were evaluated prior to biomechanical testing to rule out rotator cuff pathology. Musculotendinous junctions were sutured by using Krakow locking running stiches with No. 2 FiberWire sutures (Arthrex, Naples, FL, USA) to allow for muscle loading during testing. The humerus was transected 2 cm distal to the deltoid tuberosity.

**Figure 2:**
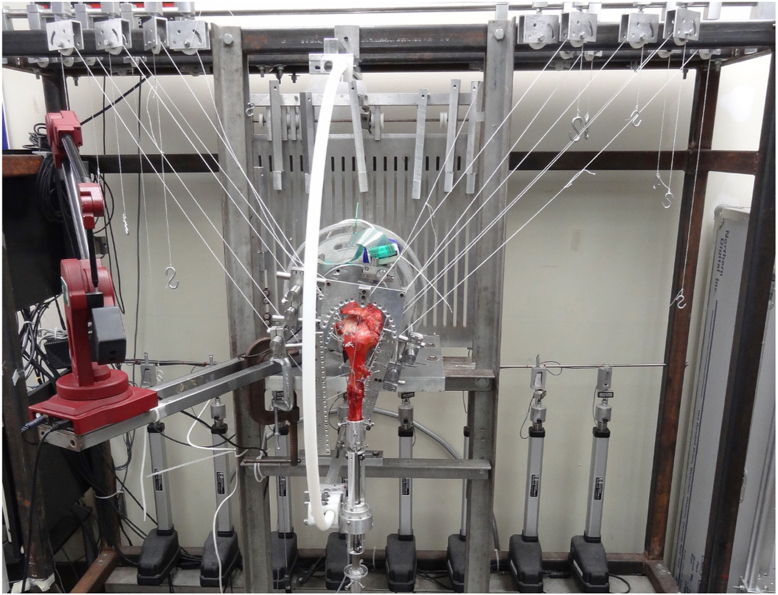
The shoulder-testing system.

The scapula was fixed to a metal plate by using 3 bolts and mounted to a custom shoulder-testing jig (Fig. 2). The scapular plate was positioned at 0° abduction and 20° anterior tilt in the sagittal plane. The transected humerus was fixed to an intramedullary rod and secured with interlocking screws. The rod was connected to a goniometer angle sensor (Novotechnik US, Southborough, MA, USA) and secured to an arc on the testing system. Once secured, the rod was positioned so that the humerus was located in the scapular plane. Humeral axial rotation was defined to be 90° external rotation when the bicipital groove aligned to the anterior edge of the acromion at 60° glenohumeral abduction. Screws were inserted in the proximal humerus at the proximal and distal aspects of the bicipital groove, coracoid, and anterior and posterior aspects of the acromion. These screws served as consistent digitizing markers for three-dimensional tracking of the location of the humerus relative to the fixed scapula and were used to confirm that the location of the acromion remained constant throughout the testing conditions.

Physiologic muscle loading during testing was simulated by using braided low-stretch Dacron fishing line (Izorline, Paramount, CA, USA) tied to the Krakow sutures at the tendinous insertions of the muscles. The lines were fed through adjustable pulleys to approximate physiologic muscle force vectors and were loaded with the desired weights. Movement of the humeral head for each testing condition was tested using 2 different loading conditions. The first loading condition simulated a balanced load and used the following muscle forces: supraspinatus, 5 N; infraspinatus, 5 N; teres minor, 5 N; subscapularis, 10 N; pectoralis major, 20 N; latissimus dorsi, 20 N; and deltoid, 40 N.^12^, ^13^, ^14^, ^15^ These forces were determined from prior muscle cross-sectional area measurements and electromyographic studies.^2^^,^^24^ The second loading condition was unbalanced and simulated a superiorly directed load; in this condition, the weights were removed from the pectoralis major and latissimus dorsi, and 40 N was added to the deltoid (80 N total).^12^, ^13^, ^14^, ^15^

### Measurements

To minimize the effect of soft tissue viscoelasticity, the specimen was preconditioned by rotating it from maximal internal humeral rotation through maximal external rotation for 5 cycles. Maximum internal and external rotations were measured under a torque of 2.2 Nm by using a torque wrench attached to the intramedullary rod. Full humeral rotational ROM was measured under balanced loading.

To evaluate the kinematics at the glenohumeral joint, the location of the humerus relative to the fixed scapula was recorded with a three-dimensional digitizing system (accuracy, 0.3 mm) (MicroScribe 3DLX; Revware, Raleigh, NC, USA). Under both balanced and unbalanced loads, the humeral head position was measured at 0°, 30°, 60°, 90°, and maximal external rotation. Each of these measurements was performed at 0°, 30°, and 60° of glenohumeral abduction in the scapular plane, which corresponded to 0°, 45°, and 90° of shoulder abduction, respectively. To evaluate shoulder stability, superior humeral head translation was calculated by comparing the change in humeral head position between the balanced and unbalanced loading conditions.

For each of the testing conditions during unbalanced loading, subacromial contact pressures were measured (maximal saturation pressure, 10.3 MPa, model 4000; Tekscan, South Boston, MA, USA). The sensor's sensitivity was set to 35, and the sensor was calibrated at 40 N and 80 N by using a load cell (model 4411; Instron, Norwood, MA, USA). After calibration, the saturation pressure (mean ± standard error of the mean) was 1.7 ± 0.3 MPa.

### Experimental conditions

**Figure 3:**
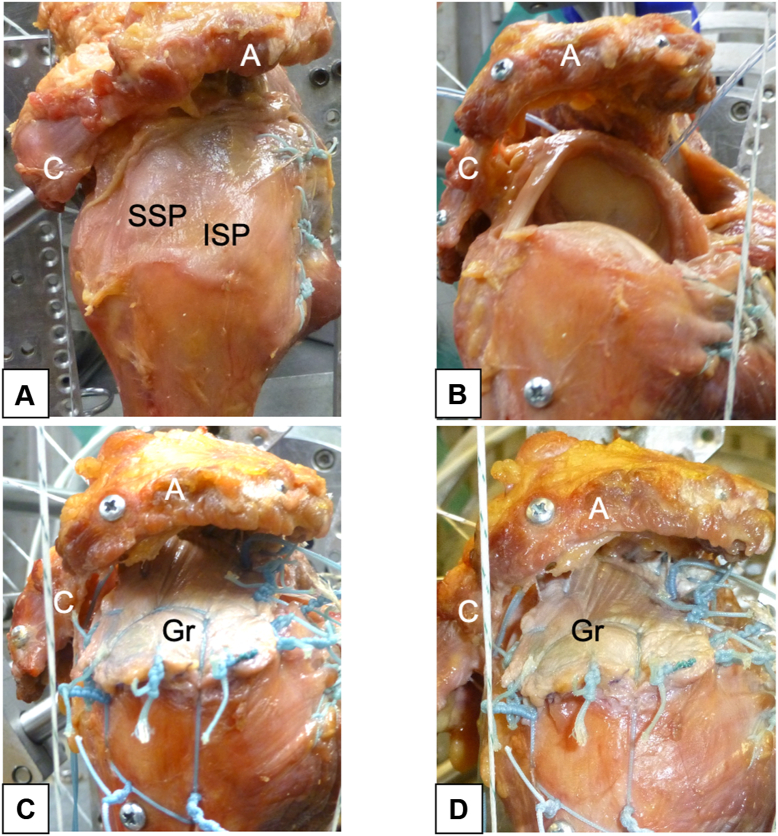
Testing conditions: (**A**) intact shoulder; (**B**) irreparable rotator cuff (supraspinatus and infraspinatus) tear; (**C**) SCR with SuturePatch augmentation performed at 20° glenohumeral abduction; (**D**) SCR with SuturePatch augmentation performed at 30° glenohumeral abduction. *A*, acromion; *C*, coracoid; *Gr*, graft; *ISP*, infraspinatus; *SSP*, supraspinatus; *SCR*, superior capsule reconstruction.

Four conditions were tested sequentially: intact shoulder; irreparable rotator cuff tear; SCR with SuturePatch augmentation performed at 20° glenohumeral abduction; and SCR with SuturePatch augmentation performed at 30° glenohumeral abduction (Fig. 3). In this study, the intact shoulder position under balanced loading was defined as the initial position. According to the 2:1 ratio rule, 20° or 30° of glenohumeral abduction corresponds to 30° or 45° of shoulder abduction, with an accompanying theoretical 10° or 15° of scapular upward rotation.^6^^,^^21^

### Irreparable rotator cuff tear

After the intact shoulder was tested, the irreparable rotator cuff tear model was created by sharply incising the supraspinatus and anterior half of the infraspinatus at their footprints on the greater tuberosity, and the lateral edges were prepared and retracted all the way to the glenoid (Fig. 3). After resection, the sizes of the defect in the anterior–posterior direction medially at the glenoid and laterally at the supraspinatus footprint were digitized at 20° of glenohumeral abduction. These measurements were then used to determine the dimensions of the fascia lata graft. For all remaining conditions, the muscle loading on the torn portions of the rotator cuff (5 N applied to the supraspinatus and 2.5 N applied to the anterior half of the infraspinatus) was removed to replicate the lack of physiologic muscle tension that occurs after an irreparable rotator cuff tear with severe muscle atrophy.^3^^,^^9^

### Superior capsule reconstruction with SuturePatch augmentation performed at 20° glenohumeral abduction

In the third condition, SCR using thin fascia lata and SuturePatch was performed at 20° glenohumeral abduction (Fig. 3). Polyester SuturePatch was used for graft augmentation (Fig. 1). SuturePatch is a reinforced polyester mesh with a thickness of approximately 2 mm. The embroidered spaces are less than 1 mm, and the fibers are interwoven in a multidirectional orientation. The stitch pattern forms an irregular, multidirectional interlaced network that enhances load sharing and minimizes stress concentration. The SuturePatch was sandwiched between 2 layers of thin fascia lata (1-2 mm thickness in each layer) (Fig. 1). The SuturePatch was securely fixed to the fascia lata by multiple No. 2 FiberWire sutures across the entire graft surface, creating a composite construct with no relative motion between the patch and the fascia lata layers. The mean thickness of the final graft was 4.4 mm (range, 4.0-4.9 mm) at the medial edge and 4.1 mm (range, 3.4-4.9 mm) at the lateral edge. Graft dimensions were calculated from the digitized measurements of the tear size. The grafts were prepared to the same size as the tear in the anterior–posterior direction. In the medial–lateral direction, the grafts were 15 mm longer than the distance between the superior glenoid to the lateral edge of the greater tuberosity footprint^16^, ^17^, ^18^ at 20° (third test condition) or 30° (fourth condition) of glenohumeral abduction.

The medial side of the graft was attached to the superior glenoid by using 2 4.5-mm metal corkscrew anchors armed with a No. 2 FiberWire, which were inserted into the superior glenoid at the 1 o'clock and 10 o'clock positions of the glenoid in the right shoulder (11 o'clock and 2 o'clock positions of the glenoid in the left shoulder). Laterally, the graft was fixed to the footprint with 2 Mason–Allen stitches and an additional horizontal mattress suture medial to them, by using No. 2 FiberWire through 2 bone tunnels in the greater tuberosity. The SuturePatch was incorporated into the fixation area both medialy at the glenoid and laterally at the greater tuberosity. In addition, 2 side-to-side sutures were performed to attach the graft posteriorly to the intact inferior half of the infraspinatus tendon.

After all measurements were performed, the lateral fixation sutures on the greater tuberosity and the posterior side-to-side sutures were cut, leaving the graft medially fixated. The transosseous technique allowed reuse of the same bone tunnels without weakening the bone while maintaining the integrity of the medial graft attachment for the retensioning of the graft at the different abduction angle in the next condition.

### Superior capsule reconstruction with SuturePatch augmentation performed at 30° glenohumeral abduction

For the fourth condition, SCR was performed again by using the same graft of thin fascia lata and SuturePatch as for the third condition (Fig. 3). However, in the fourth condition, the shoulder was abducted to 30° of glenohumeral abduction prior to lateral retensioning of the graft. The reconstruction followed the same surgical technique as for the third condition.

### Data analysis and statistics

In all testing conditions, each measurement was performed in duplicate and then averaged. The average values across all 8 specimens were used for data analysis. Two-way repeated-measures analysis of variance was used to compare among the 4 testing conditions. When a difference was statistically significant, Tukey post hoc testing was used to determine testing conditions that differed (version 6.0 STATISTICA; StatSoft, Tulsa, OK, USA). Data are presented as means ± standard error of the mean, and the significance level was set at *P* < .05.

To determine the appropriate sample size, a power analysis was performed by using the G∗Power3 statistical analysis software package. Power (1 − β) was calculated by defining the sample size as 8, the level of significance (α) as 0.05, and the effect size as 0.48-4.06 for superior translation and 0.36-8.82 for subacromial peak contact pressure. The power analysis indicated that a total sample size of 8 specimens provided 80% power (1 − β = 0.8; α = 0.05) to detect significant differences between 8 specimens, assuming a power of 0.99-1.00.

## Results

### Superior stability

Compared with the intact condition, creation of an irreparable supraspinatus and infraspinatus tendon tear significantly increased superior translation from 0° to 90° external rotation at 0° (*P* < .001) and 30° (*P* < .001) glenohumeral abduction. Compared with the condition of irreparable supraspinatus and infraspinatus tendon tear, SCR with SuturePatch augmentation performed at both 20° (*P* < .05) and 30° (*P* < .01) glenohumeral abduction significantly decreased superior translation in all rotated and abducted positions. Superior translation after SCR with SuturePatch augmentation performed at 30° glenohumeral abduction did not differ significantly from that of the intact condition at all positions (29%-175% relative to intact condition, *P* = .08-.98). For SCR performed at 20° glenohumeral abduction, superior translation did not differ from that in the intact condition except at 60° external rotation in 0° abduction (217% relative to intact condition, *P* = .04) and at 0° external rotation in 30° abduction (176% relative to intact condition, *P* = .04). Direct comparison between 20° and 30° glenohumeral abduction for performing SCR with SuturePatch augmentation showed no significant difference in superior translation in any position (*P* = .43-.99) (Table I).

**Table I:** Superior glenohumeral translation.

Measurement position	Intact		Irreparable supraspinatus–infraspinatus tear		SCR with SuturePatch augmentation performed at 20° GH abduction		SCR with SuturePatch augmentation performed at 30° GH abduction	
	Translation (mm)	% Translation	Translation (mm)	% Translation	Translation (mm)	% Translation	Translation (mm)	% Translation
0° GH abduction								
0° ER	2.3 ± 0.3	100%	7.8 ± 0.8∗	339%	3.3 ± 0.6†	143%	2.6 ± 0.5†	113%
30° ER	1.3 ± 0.2	100%	6.5 ± 0.6∗	500%	2.6 ± 0.5†	200%	2.2 ± 0.3†	169%
60° ER	1.2 ± 0.2	100%	6.4 ± 0.6∗	533%	2.6 ± 0.5∗^,^†	217%	2.1 ± 0.3†	175%
90° ER	1.5 ± 0.4	100%	5.6 ± 0.8∗	373%	2.6 ± 0.6†	173%	1.8 ± 0.4†	120%
30° GH abduction								
0° ER	1.7 ± 0.2	100%	5.8 ± 0.6∗	341%	3.0 ± 0.6∗^,^†	176%	2.9 ± 0.3†	171%
30° ER	1.4 ± 0.1	100%	4.9 ± 0.7∗	350%	1.6 ± 0.5†	114%	2.0 ± 0.4†	143%
60° ER	1.1 ± 0.3	100%	4.4 ± 0.6∗	400%	1.5 ± 0.5†	136%	1.6 ± 0.4†	145%
90° ER	1.3 ± 0.3	100%	4.1 ± 0.7∗	315%	2.1 ± 0.6†	162%	1.8 ± 0.4†	138%
60° GH abduction								
0° ER	2.3 ± 0.1	100%	3.5 ± 0.4	152%	1.5 ± 0.2†	65%	0.8 ± 0.4†	35%
30° ER	1.3 ± 0.5	100%	2.1 ± 0.6	162%	0.9 ± 0.5†	69%	0.6 ± 0.3†	46%
60° ER	0.7 ± 0.2	100%	1.1 ± 0.2	157%	0.4 ± 0.3†	57%	0.2 ± 0.3†	29%
90° ER	1.1 ± 0.3	100%	1.5 ± 0.3	136%	0.7 ± 0.3†	64%	0.5 ± 0.2†	45%

*ER*, external rotation; *GH*, glenohumeral; *SCR*, superior capsule reconstruction.

Values are given as mean ± standard error. The % superior translation was calculated by dividing each value by that for the intact condition at the same position.

∗ Value significantly (*P* < .05) different from that for the intact condition (condition 1).

† Value significantly (*P* < .05) different from that for simulated supraspinatus and infraspinatus tear (condition 2).

### Subacromial peak contact pressure

Compared with the intact condition, irreparable supraspinatus and infraspinatus tendon tear significantly increased subacromial peak contact pressure at 0° (*P* < .001), 30° (*P* < .001), and 60° (*P* = .01) external rotation in 0° glenohumeral abduction and at 30° (*P* < .001), 60° (*P* = .008), and 90° (*P* = .005) external rotation in 30° glenohumeral abduction. Compared with the condition of irreparable supraspinatus and infraspinatus tendon tear, SCR with SuturePatch augmentation performed at both 20° and 30° glenohumeral abduction significantly decreased subacromial peak contact pressure from 0° to 90° external rotation in 0° glenohumeral abduction (*P* < .001) and at 30° (*P* < .001) and 90° (*P* < .05) external rotation in 30° glenohumeral abduction. After SCR with SuturePatch augmentation performed at both 20° and 30° glenohumeral abduction, subacromial peak contact pressure was similar to that of the intact condition all rotation and abduction positions. Direct comparison between 20° and 30° glenohumeral abduction for performing SCR with SuturePatch augmentation showed no significant difference in subacromial peak contact pressure at any position (*P* = .61-.99) (Table II).

**Table II:** Subacromial peak contact pressure.

Measurement position	Intact		Irreparable supraspinatus–infraspinatus tear		SCR with SuturePatch augmentation performed at 20° GH abduction		SCR with SuturePatch augmentation performed at 30° GH abduction	
	Pressure (MPa)	% Pressure	Pressure (MPa)	% Pressure	Pressure (MPa)	% Pressure	Pressure (MPa)	% Pressure
0° GH abduction								
0° ER	0.35 ± 0.05	100%	1.12 ± 0.10∗	320%	0.18 ± 0.02†	51%	0.16 ± 0.06†	46%
30° ER	0.56 ± 0.06	100%	1.08 ± 0.10∗	193%	0.36 ± 0.05†	64%	0.26 ± 0.05†	46%
60° ER	0.64 ± 0.06	100%	1.10 ± 0.08∗	172%	0.35 ± 0.05†	55%	0.24 ± 0.04†	38%
90° ER	0.48 ± 0.07	100%	0.73 ± 0.12	152%	0.25 ± 0.06†	52%	0.26 ± 0.06†	54%
30° GH abduction								
0° ER	0.40 ± 0.11	100%	0.63 ± 0.17	158%	0.45 ± 0.06	113%	0.33 ± 0.06	83%
30° ER	0.42 ± 0.07	100%	1.19 ± 0.13∗	283%	0.53 ± 0.07†	126%	0.44 ± 0.11†	105%
60° ER	0.28 ± 0.04	100%	0.64 ± 0.10∗	229%	0.39 ± 0.05	139%	0.41 ± 0.09	146%
90° ER	0.25 ± 0.04	100%	0.61 ± 0.10∗	244%	0.32 ± 0.03†	128%	0.23 ± 0.04†	92%
60° GH abduction								
0° ER	0.09 ± 0.04	100%	0.20 ± 0.11	222%	0.11 ± 0.04	122%	0.16 ± 0.07	178%
30° ER	0.24 ± 0.10	100%	0.32 ± 0.10	133%	0.21 ± 0.06	88%	0.33 ± 0.08	138%
60° ER	0.24 ± 0.07	100%	0.26 ± 0.08	108%	0.24 ± 0.07	100%	0.20 ± 0.05	83%
90° ER	0.08 ± 0.03	100%	0.09 ± 0.03	113%	0.13 ± 0.05	163%	0.13 ± 0.04	163%

*ER*, external rotation; *GH*, glenohumeral; *SCR*, superior capsule reconstruction.

Values are given as mean ± standard error. The % superior translation was calculated by dividing each value by that for the intact condition at the same position.

∗ Value significantly (*P* < .05) greater than that for the intact condition (condition 1).

† Value significantly (*P* < .05) less than that for simulated supraspinatus and infraspinatus tear (condition 2).

### Glenohumeral rotational range of motion

Compared with the intact condition, creation of an irreparable supraspinatus and infraspinatus tendon tear significantly increased total rotational ROM at 0° (by 9°, *P* = .01), 30° (by 10°, *P* = .004), and 60° (by 18°, *P* < .001) glenohumeral abduction. SCR with SuturePatch augmentation performed at both 20° and 30° glenohumeral abduction did not change internal rotation (*P* = .39-.99), external rotation (*P* = .61 to 0.99), or total rotational ROM (*P* = .51-.99) compared with those during irreparable supraspinatus and infraspinatus tendon tear at all rotation and abduction positions (Table III).

**Table III:** Rotational range of motion.

	Intact	Irreparable supraspinatus–infraspinatus tear	SCR with SuturePatch augmentation performed at 20° GH abduction	SCR with SuturePatch augmentation performed at 30° GH abduction
0° GH abduction				
Internal rotation	17 ± 4	24 ± 5	21 ± 4	20 ± 5
External rotation	105 ± 4	107 ± 4∗	108 ± 4∗	108 ± 4
Total rotational ROM	122 ± 8	131 ± 8∗	128 ± 8	127 ± 8
30° GH abduction				
Internal rotation	16 ± 5	22 ± 5	22 ± 4	23 ± 4
External rotation	115 ± 2	118 ± 2	118 ± 2	120 ± 2∗
Total rotational ROM	131 ± 5	141 ± 6∗	140 ± 5∗	142 ± 5∗
60° GH abduction				
Internal rotation	0 ± 5	13 ± 7∗	10 ± 6∗	11 ± 6∗
External rotation	114 ± 3	119 ± 3∗	120 ± 2∗	121 ± 2∗
Total rotational ROM	114 ± 6	132 ± 7∗	130 ± 6∗	132 ± 6∗

*GH*, glenohumeral; *ROM*, range of motion; *SCR*, superior capsule reconstruction.

Values are given as mean ± standard error.

∗ Value significantly (*P* < .05) different from value for intact condition.

## Discussion

Recent reports have shown that SCR improves shoulder function in patients with irreparable rotator cuff tears.^1^^,^^4^^,^^5^^,^^7^^,^^8^^,^^10^^,^^11^^,^^16^, ^17^, ^18^^,^^22^ However, most reports have relatively high rate of the graft tear,^5^^,^^10^^,^^11^ which is correlated with decreased clinical outcome after SCR^16^^,^^18^ A previous biomechanical study showed that increasing the SCR graft thickness improves superior glenohumeral stability, and fascia lata grafts thinner than 4 mm only partially restored superior stability after SCR.^13^ In some cases, fascia lata autografts thicker than 4 mm cannot be achieved due to variability in the thickness of the fascia lata in each patient. For these cases, SuturePatch was developed to reinforce the graft for SCR. In this study, SCR with SuturePatch augmentation significantly decreased superior translation to the intact level even though the fascia lata was only 2-3 mm thick. This result suggests that the SuturePatch can reinforce thin fascia lata grafts to restore superior stability completely.

In the current study, the optimal tension of SuturePatch was assessed by attaching it to the glenoid and greater tuberosity during SCR surgery at different glenohumeral abduction angles. High glenohumeral abduction during SCR increases the tension of the graft.^13^ A previous biomechanical study showed that SCR performed at 30° glenohumeral abduction, which represents 45° shoulder abduction, completely restored superior glenohumeral translation,^12^ whereas a clinical study reported that SCR performed at 45° shoulder abduction led to postoperative shoulder stiffness in some patients.^16^ These results suggest that glenohumeral abduction exceeding 30° makes the graft too tight for optimal SCR. Therefore, we chose 30° glenohumeral abduction as one of the surgical positions evaluated in the current study. In addition, a recent clinical study recommended 30° shoulder abduction for SCR using fascia lata autografts to restore superior shoulder stability without postoperative shoulder stiffness.^17^^,^^18^ Furthermore, a biomechanical study showed that SCR using fascia lata completely restored superior stability of the shoulder joint when the graft was attached at 10°-30° glenohumeral abduction.^13^ From these previous studies, we selected 20° glenohumeral abduction, which represents 30° shoulder abduction, for comparison of superior glenohumeral translation and subacromial peak contact pressure with 30° glenohumeral abduction. In the current study, the average superior translation and subacromial peak contact pressure in SCR performed at 30° glenohumeral abduction were less than those at 20° glenohumeral abduction. However, the difference (less than 0.8 mm in superior translation and less than 0.12 MPa in subacromial peak contact pressure) was not statistically significant. Therefore, 30°-45° of shoulder abduction may provide the optimal tension for SCR when SuturePatch is used for augmentation of the graft.

In the current study, we measured the glenohumeral rotational ROM to assess the possibility of postoperative shoulder stiffness. At both 20° and 30° glenohumeral abduction, SCR with SuturePatch augmentation did not significantly decrease external rotation, internal rotation, and total rotational ROM compared with before SCR, suggesting that SuturePatch augmentation might not cause postoperative shoulder stiffness after SCR. The possible explanation for this benefit is the structure of SuturePatch. Because it is a mesh, SuturePatch can extend during glenohumeral rotation to adapt to fascia lata graft and residual shoulder capsule, even though it is made from hard polyester. Furthermore, SuturePatch can extend like a hammock when the humeral head is translated superiorly, as do the shoulder capsular ligaments.^20^^,^^23^ Therefore, SCR with SuturePatch augmentation both completely restored superior glenohumeral stability and maintained glenohumeral ROM.

Strengths of this cadaveric biomechanical study included direct measurement of superior translation, subacromial peak contact pressure, and ROM in the glenohumeral joint; these parameters cannot be investigated in living subjects. In addition, we measured superior translation, subacromial contact pressure, and glenohumeral ROM under 4 conditions by using the same specimens; these conditions would not have been possible in vivo.

Our study had several weaknesses. First, muscle loading was static rather than dynamic because of the cadaveric materials used. Second, we tested only fascia lata grafts. We plan to assess the effects of SuturePatch augmentation by using another graft material, such as dermal allografts or xenografts, in the near future.

## Conclusion

SCR with SuturePatch augmentation restored superior glenohumeral stability and maintained glenohumeral ROM. When SuturePatch is used for graft augmentation during SCR, 30° or 45° of shoulder abduction (equal to 20° or 30° of glenohumeral abduction) may provide the optimal tension for graft attachment to the glenoid medially and greater tuberosity laterally.

## Disclaimers:

Funding: No funding was disclosed by the authors.

Conflicts of interest: The authors, their immediate families, and any research foundations with which they are affiliated have not received any financial payments or other benefits from any commercial entity related to the subject of this article.
